# Combined superior mesenteric artery syndrome and nutcraker syndrome presenting as acute pancreatitis: a case report

**DOI:** 10.1590/1677-5449.202201612

**Published:** 2023-06-16

**Authors:** Bárbara Neto Castro, Ana Rita Ferreira, Susana Graça, Manuel Oliveira

**Affiliations:** 1 Centro Hospitalar Vila Nova de Gaia/Espinho - CHVNG/E, Vila Nova de Gaia, Porto, Portugal.

**Keywords:** superior mesenteric artery syndrome, nutcracker syndrome, acute pancreatitis, rare diseases, síndrome da artéria mesentérica superior, síndrome de quebra-nozes, pancreatite aguda, doenças raras

## Abstract

Superior mesenteric artery syndrome designates compression of the third part of the duodenum between the superior mesenteric artery and the aorta. This condition has a low incidence, being more common in thin young women. Nutcracker syndrome is compression of the left renal vein between the superior mesenteric artery and the aorta. Both entities are rare, and their coexistence has been reported in a few cases. Conservative treatment targeting weight gain is sufficient in most cases. An association between the superior mesenteric artery syndrome and acute pancreatitis has rarely been reported. We intend to describe the case of an 18-year-old girl who was admitted to the emergency room with epigastric pain and emesis. Our investigation revealed acute acalculous pancreatitis. During work-up, we discovered superior mesenteric artery syndrome and a compressed left renal vein. The patient is on conservative treatment, and her symptoms have improved.

## Resumo

A síndrome da artéria mesentérica superior designa compressão da terceira parte do duodeno pela artéria mesentérica superior e a aorta. Essa condição tem uma baixa incidência, sendo mais comum em mulheres jovens magras. A síndrome de quebra-nozes resulta da compressão da veia renal esquerda pela artéria mesentérica superior e a aorta. Ambas as entidades são raras, e a sua coexistência foi descrita em poucos casos. Tratamento conservador com o objetivo de ganho ponderal é suficiente na maioria dos casos. A associação entre a síndrome da artéria mesentérica superior e a pancreatite aguda foi raramente relatada. Pretendemos descrever o caso de uma jovem de 18 anos que recorreu ao serviço de urgência com epigastralgia e vômitos. A investigação realizada revelou pancreatite aguda alitiásica. Durante o estudo complementar, foi identificada a síndrome da artéria mesentérica superior, bem como uma veia renal esquerda comprimida. A paciente encontra-se em tratamento conservador, apresentando melhora clínica.

## INTRODUCTION

Superior mesenteric artery (SMA) syndrome (also known as Wilkie Syndrome) designates compression of the third part of the duodenum between the SMA and the aorta, causing symptoms such as post-prandial abdominal pain, vomiting, abdominal fullness, sub-occlusive crisis, and nausea.^1,2^ SMA syndrome is rare, with an incidence between 0.13% and 0.3%, is most common in thin young females, and is associated with weight loss that reduces fat at the aortomesenteric angle.^1^

Nutcracker (NC) syndrome is compression of the left renal vein between the SMA and the aorta. It is also rare and may course with left flank pain with or without hematuria, proteinuria, renal insufficiency, and varicocele.^1-3^

Both diagnoses are made with a combination of imaging findings and clinical presentation.^1^ First line treatment focuses on weight gain with nutritional support combined with prokinetic and antiemetic agents.^1,4^

We aim to report the clinical case of a young female with both SMA syndrome and NC syndrome, presenting as acute acalculous pancreatitis. Herein, we discuss the diagnosis and management of this rare combination of conditions to contribute further details to the current state of the art.

This manuscript complies with the Helsinki Declaration and with local ethical guidelines. Written informed consent was obtained from the patient for publication of this case report.

## CASE REPORT

An 18-year-old, previously healthy female with a body mass index of 15.2 kg/m^2^ presented at the emergency department with epigastric pain and emesis, with onset 24 hours previously. The patient did not have a history of marked weight loss, but referred to easy weight loss in times of anxiety. On examination, she presented tenderness to palpation of the epigastric region. The lab work-up revealed normal liver function, mildly raised C-reactive protein (1.29 mg/dL [Reference range (RR): 0-0.5 mg/dL]), amylase of 570 U/L [Reference range (RR): 13-53 U/L ], and lipase of 1478 U/L [Reference range (RR): 13-60 U/L ]. Abdominal ultrasound did not reveal any changes. The patient was treated conservatively and the cause of her pancreatitis was investigated. The most common causes of pancreatitis, such as alcoholic pancreatitis, hypertriglyceridemia, hypercalcemia, and drug-induced pancreatitis, were excluded. Magnetic resonance cholangiopancreatography revealed slight liquid distension of the first and second portions of the duodenum, suggesting superior mesenteric artery syndrome. Her symptoms subsided and she was discharged after 6 days and advised to attend a follow-up appointment.

At the first medical consultation, 1 month after discharge, the patient still presented with nausea, occasional epigastric pain, early satiety, and postprandial fullness. The patient maintained her initial weight. The lab work-up, namely pancreatic enzyme tests, revealed no abnormalities. We prescribed metoclopramide and made dietetic recommendations.

As we continued with the clinical investigation, an auto-immune basis was excluded and work-up over the following months revealed coexistence of the SMA syndrome and the NC Syndrome.

Upper gastrointestinal series/upper gastrointestinal double contrast radiograph showed a markedly hypotonic, slightly hypokinetic stomach with normal morphology and topography, evacuating at a satisfactory rate.

Upper digestive endoscopy revealed distension of the second portion of the duodenum and mild chronic gastritis that we treated with a proton pump inhibitor.

**Figure 1:**
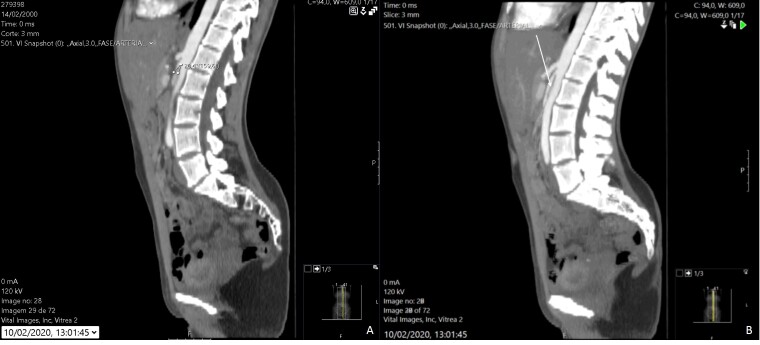
Saggital plane of an abdominal CT showing a decreased aortomesenteric angle (A), causing compression of the left renal vein (white arrow) (B).

**Figure 2:**
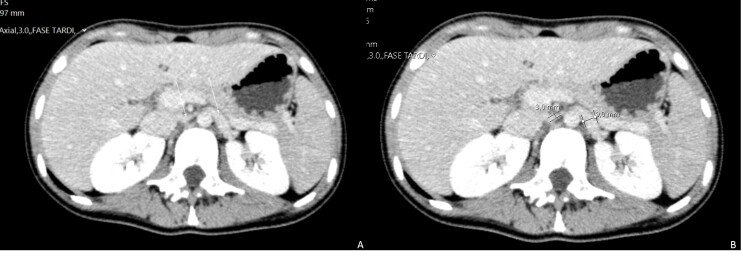
Axial plane of abdominal CT showing stenosis of the left renal vein (white arrows) between the aorta and the superior mesenteric artery (A); a significantly decreased left renal vein caliber is evident (B).

**Figure 3:**
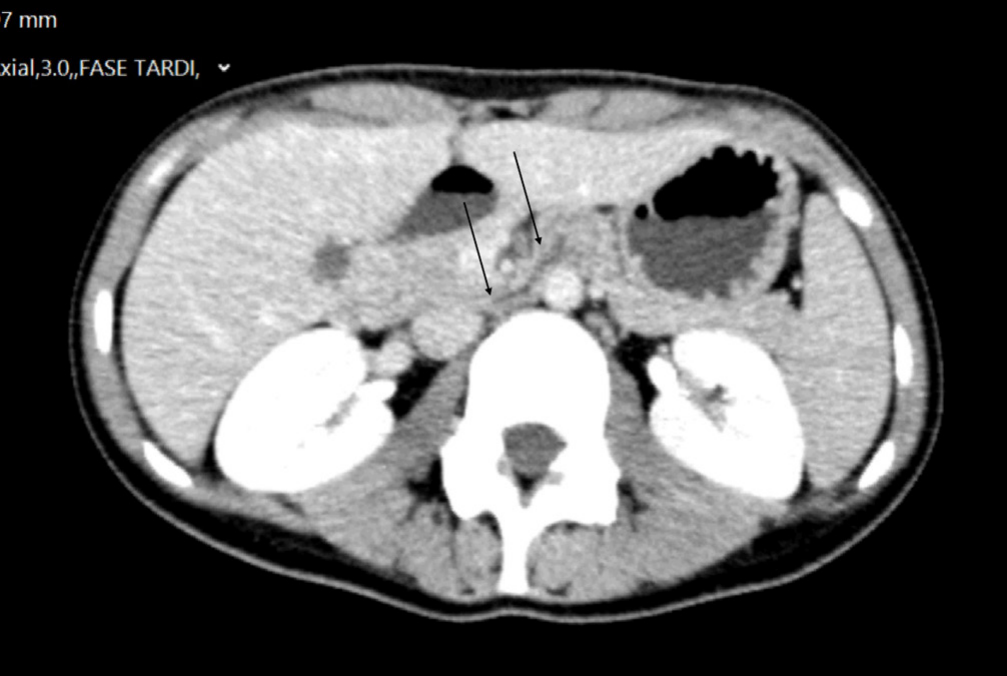
Axial plane of abdominal CT showing narrowing of the duodenum (black arrows) between the aorta and the superior mesenteric artery.

Abdominopelvic CT (AP-CT) revealed signs of SMA syndrome: decreased space between the SMA and the aorta (5mm distance and a 20º angle), distended stomach and duodenum until the clamp zone, and decreased caliber of the third portion of the duodenum (4mm). AP-CT also revealed findings compatible with NC syndrome: significantly decreased caliber of the left renal vein, with a caliber of 3mm in the aortomesenteric space and the rest of the renal vein from the hilum to the compression site with a prominent caliber of 9 mm (Figures 1, 2, and 3).

During follow-up, the patient also complained of left lumbar/flank pain, which she self-medicated with analgesics.

It was decided to refer the patient for psychiatric evaluation to rule out eating disorders and she was referred for nutritional support, for weight optimization. She was seen by a nutritionist who prepared personalized nutritional plans from 2100 to 2400 Kcal/day in a liquid diet.

Over 2 years of follow-up the patient has experienced several weight variations. At the moment, her weight is stable (BMI 16 kg/m^2^) and her symptoms have improved.

## DISCUSSION

Both SMA syndrome and NC syndrome are caused by the entrapment effect of clamping between the abdominal aorta and the SMA. Weight loss is a common etiology because it reduces the fat plane between both vessels.

Both entities are rare, and their coexistence had been reported in only a few cases. In 2017, Oh^5^ summarized 8 cases of combined SMA syndrome with NC syndrome, including their own novel description.^5^ During our research we found nearly 20 additional cases.^1-4,6-17^

Our patient was the typical thin young female, although SMA syndrome may also occur in men and in all age groups.^1^ The diagnosis of SMA syndrome is based on the clinical setting and with the help of imaging modalities such as CT or MRI scans that may show a dilated duodenum proximal to the clamp, a short aortomesenteric distance, or a narrow aortomesenteric angle.^1^ A normal aortomesenteric angle remains between 28 and 65 degrees and a normal aortomesenteric distance is between 10 and 34 mm.^18^ Our patient has a 20º aortomesenteric angle and an aortomesenteric distance of 5mm.

Conservative treatment for weight gain is sufficient in most cases, starting with enteric fluids if the patient is able to tolerate them. Treatment with nasojejunal or jejunostomy feeding tubes or parenteral nutrition may also be considered. Nonetheless, some series have reported conservative treatment failure rates of 50-70%.^19^ In cases where surgical treatment is needed, options are duodenojejunostomy, gastrojejunostomy, and Strong’s procedure (mobilization of the duodenum by dividing the ligament of Treitz, the duodenum is then positioned to the right of the SMA).^1^

NC syndrome can be diagnosed by Doppler, ultrasound, CT or MRI scan, or with an invasive, but conclusive, left renal vein phlebography.^1,20^ The first line treatment is the same as for SMA syndrome. A variety of surgical and endovascular interventions are available for refractory cases.^1,2^

The association between SMA syndrome and acute pancreatitis is rarely reported.^21-25^ SMA syndrome may produce retrograde bile reflux into the pancreatic duct, which activates the inflammatory process of pancreatitis, but this remains a controversial issue.^24,25^ We believe that acute pancreatitis was caused by the SMA since no other cause was found and the pancreatitis resolved, but the patient maintained symptoms attributable to SMA.

Our patient presented with epigastric pain and vomiting, so SMA syndrome was not high on our list of differential diagnoses. In our list of possible diagnoses, we included gastroenteritis, peptic ulcer disease, cholelithiasis, pancreatitis and, though less likely considering her age, bowel obstruction. The initial work-up revealed an acute acalculous pancreatitis and a suspicion of SMA syndrome. We believe, as some authors suggest, that this association may be related to a retrograde reflux of bile. In 2019, Wang et al.^25^ described the case of a 19-year-old female with SMA syndrome, NC syndrome, pancreatitis, and gallbladder distention.^25^ Therefore, to the best of our knowledge, this is the second case where SMA syndrome, NC syndrome, and pancreatitis coexist. The patient also complained of occasional left lumbar/flank pain, compatible with NC syndrome, but did not show any other symptoms. The rest of the work-up confirmed SMA syndrome and NC syndrome, and conservative management with nutritional support and prokinetic and antiemetic agents was started, which resulted in symptom improvement. We do not have more recent images of the patient, which constitutes a limitation in the description of this case. Given the patient's symptomatic improvement, unfortunately we have constraints to ordering imaging exams in this situation, although academically it would be of great interest.

## CONCLUSION

SMA syndrome has a low incidence and is more common in young women with low weight. The combination with NC syndrome is rare, despite the shared common etiological mechanism. Conservative treatment focused on weight gain is sufficient in most cases, allied with prokinetic and antiemetic agents. Surgical options and endovascular stenting are available for refractory cases, but no gold-standard has been defined.

The association between the SMA syndrome and acute pancreatitis has been described in less than ten cases and may be related to a retrograde reflux of bile. This is an extremely rare case due to the association of these three conditions. To the best of our knowledge, it is the second case ever reported.
